# hBMSC-Derived Extracellular Vesicles Attenuate IL-1β-Induced Catabolic Effects on OA-Chondrocytes by Regulating Pro-inflammatory Signaling Pathways

**DOI:** 10.3389/fbioe.2020.603598

**Published:** 2020-12-14

**Authors:** Shushan Li, Sabine Stöckl, Christoph Lukas, Julia Götz, Marietta Herrmann, Marianne Federlin, Susanne Grässel

**Affiliations:** ^1^Department of Orthopaedic Surgery, Experimental Orthopaedics, Centre for Medical Biotechnology (ZMB/Biopark 1), University of Regensburg, Regensburg, Germany; ^2^Department of Orthopaedic Surgery, Asklepiosklinikum, Bad Abbach, Germany; ^3^Interdisciplinary Center for Clinical Research (IZKF) Group Tissue Regeneration in Musculoskeletal Diseases, Bernhard-Heine-Centrum for Locomotion Research, University Hospital Würzburg, University of Würzburg, Würzburg, Germany; ^4^Department of Conservative Dentistry and Periodontology, University Medical Center Regensburg, Regensburg, Germany

**Keywords:** extracellular vesicles, IL-1ß, osteoarthritis, signaling pathways, hBMSC, chondrocytes

## Abstract

**Background:** Human bone marrow-derived mesenchymal stromal cells (hBMSCs) provide a promising therapeutic approach in the cell-based therapy of osteoarthritis (OA). However, several disadvantages evolved recently, including immune responses of the host and regulatory hurdles, making it necessary to search for alternative treatment options. Extracellular vesicles (EVs) are released by multiple cell types and tissues into the extracellular microenvironment, acting as message carriers during intercellular communication. Here, we investigate putative protective effects of hBMSC-derived EVs as a cell-free approach, on IL-1β-stimulated chondrocytes obtained from OA-patients.

**Methods:** EVs were harvested from the cell culture supernatant of hBMSCs by a sequential ultracentrifugation process. Western blot, scanning electron microscopy (SEM), and nanoparticle tracking analysis (NTA) were performed to characterize the purified particles as EVs. Intracellular incorporation of EVs, derived from PHK26-labeled hBMSCs, was tested by adding the labeled EVs to human OA chondrocytes (OA-CH), followed by fluorescence microscopy. Chondrocytes were pre-stimulated with IL-1β for 24 h, followed by EVs treatment for 24 h. Subsequently, proliferation, apoptosis, and migration (wound healing) were analyzed via BrdU assay, caspase 3/7 assay, and scratch assay, respectively. With qRT-PCR, the relative expression level of anabolic and catabolic genes was determined. Furthermore, immunofluorescence microscopy and western blot were performed to evaluate the protein expression and phosphorylation levels of Erk1/2, PI3K/Akt, p38, TAK1, and NF-κB as components of pro-inflammatory signaling pathways in OA-CH.

**Results:** EVs from hBMSCs (hBMSC-EVs) promote proliferation and reduce apoptosis of OA-CH and IL-1β-stimulated OA-CH. Moreover, hBMSC-EVs attenuate IL-1β-induced reduction of chondrocyte migration. Furthermore, hBMSC-EVs increase gene expression of PRG4, BCL2, and ACAN (aggrecan) and decrease gene expression of MMP13, ALPL, and IL1ß in OA-CH. Notably, COL2A1, SOX9, BCL2, ACAN, and COMP gene expression levels were significantly increased in IL-1β^+^ EV groups compared with those IL-1β groups without EVs, whereas the gene expression levels of COLX, IL1B, MMP13, and ALPL were significantly decreased in IL-1β^+^ EV groups compared to IL-1β groups without EVs. In addition, the phosphorylation status of Erk1/2, PI3K/Akt, p38, TAK1, and NF-κB signaling molecules, induced by IL-1β, is prevented by hBMSC- EVs.

**Conclusion:** EVs derived from hBMSCs alleviated IL-1β-induced catabolic effects on OA-CH via promoting proliferation and migration and reducing apoptosis, probably via downregulation of IL-1ß-activated pro-inflammatory Erk1/2, PI3K/Akt, p38, TAK1, and NF-κB signaling pathways. EVs released from BMSCs may be considered as promising cell-free intervention strategy in cartilage regenerative medicine, avoiding several adverse effects of cell-based regenerative approaches.

## Introduction

Osteoarthritis (OA) is one of the most common age-related degenerative disorders of the joints, mostly prevalent in the joints of the hip and knee (Silverwood et al., 2015), which has a significant negative effect on the quality of life of OA-affected individuals (Glyn-Jones et al., 2015). Conventional treatment of OA includes pharmacological drug management, physical therapy, arthroscopic surgery, and eventually joint replacement with artificial prostheses (Gregori et al., 2018). Even though surgical treatment may attenuate pain and increase the quality of life of end-stage patients, the risks of infection, revision surgery, and other implant-related complications is indisputable, especially in elderly patients (Bereza et al., 2013; Khan et al., 2015). For that, it would be desirable to identify novel therapeutic approaches to protect cartilage and other joint tissues from damage and thus slow down OA progression.

Human bone marrow-derived mesenchymal stromal cells (hBMSCs) have been widely investigated and constitute a promising therapeutic approach in the cell-based therapy of cartilage trauma and thereof resulting OA (Kristjánsson and Honsawek, 2014). Several previous clinical trials reported that autologous hBMSCs used to treat cartilage defects and OA results in alleviation of pain and improvement of joint function (Wakitani et al., 2002; Matsumoto et al., 2010). Factors derived from hBMSCs promote cartilage regeneration, proliferation of OA chondrocytes (OA-CH), and synthesis of components to stabilize and restore the extracellular cartilage matrix (Wu et al., 2011; Lai et al., 2013; Toh et al., 2014; Hofer and Tuan, 2016). However, it becomes more and more evident that not the cells but their secretome components are responsible for the observed effects on tissue regeneration. This observation plus tedious regulatory hurdles make it necessary to search for alternative treatment options avoiding the cells but focusing on their secretome (Lee et al., 2010; Hwang et al., 2012; Toh et al., 2014).

Extracellular vesicles (EVs) are enclosed by a lipid bilayer, ranging between 30 and 5,000 nm in diameter, and exosomes, one subgroup of EVs that are small (50–150 nm) extracellular membrane-covered vesicles with a complex cargo of nucleic acids, proteins, and lipids, are released by multiple cell types and tissues into the extracellular microenvironment (Zhang et al., 2019), acting as a kind of molecular message carriers in intercellular communication. Different types of vesicles can be distinguished based on their biogenesis route. Invagination of vesicles in multivesicular bodies (MVBs) and their release after fusion of MVBs with the plasma membrane are the precursors of EVs (Théry et al., 2006). Due to overlapping size ranges and limited specificity of current enrichment and analyses methods, MVs and exosomes can, in most of the cases, not be distinguished and are both often referred to as small EVs. Numerous preclinical *in vivo* and *in vitro* studies demonstrated that EVs modulate multiple biological processes including immune response and inflammation (Mobergslien and Sioud, 2014; Lian et al., 2017; Salvi et al., 2018, p. 7).

EVs derived from mesenchymal stromal cells (MSCs) have been analyzed in preclinical *in vivo* small animal studies and *in vitro* cell culture studies but have not yet progressed to application in clinical trials (de Araujo Farias et al., 2018; Wang et al., 2020; Zhao et al., 2020). Recently, some studies have demonstrated that EVs derived from MSCs attenuated inflammatory tissue reactions during OA pathogenesis (Zhang et al., 2018, 2019). However, the underlying molecular mechanisms on how EVs modulate inflammatory processes in OA pathology need more attention.

It is well-known that IL-1β, as an important inflammatory mediator, plays a crucial role in the progression of OA (Daheshia and Yao, 2008; Han et al., 2018; Jenei-Lanzl et al., 2019). In line with that knowledge, we used IL-1β stimulation of articular chondrocytes as a standard *in vitro* OA model to induce catabolic effects resembling metabolic changes in OA pathology. Subsequently, we investigated the putative protective effects of hBMSC-derived EVs (hBMSC-EVs), as a cell-free approach, on IL-1β-stimulated chondrocytes obtained from OA patients.

**Figure 1:**
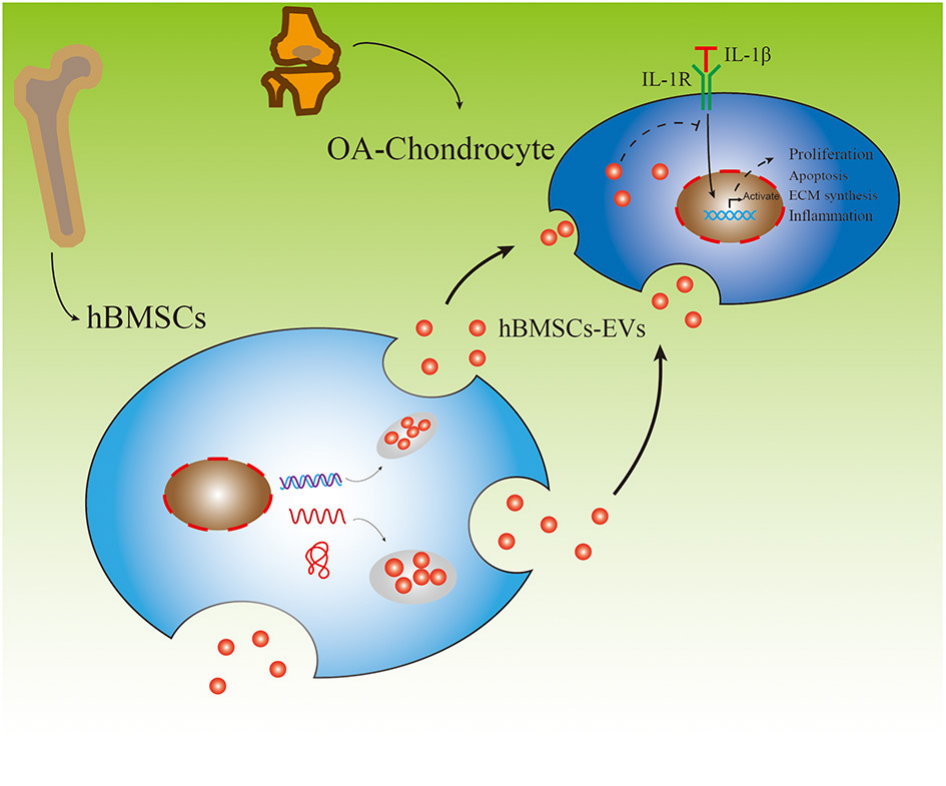
Experimental outline of responses of IL-1β-induced osteoarthritic chondrocytes (OA-CH) treated with hBMSC-derived EVs (hBMSC-EVs).

The aim of this study was to evaluate the effect of hBMSC-derived EVs on OA-chondrocytes (OA-CH). hBMSC and OA-CH were obtained from patients who underwent hip and knee arthroplasty surgery, respectively. OA-CH were stimulated with IL-1β to establish a standard *in vitro* OA model. hBMSC-EVs were used as a cell-free therapeutic approach to treat IL-1β-stimulated OA-CH. We hypothesized that hBMSC-EVs could reverse effects induced by IL-1β on OA-CH, such as activation of inflammatory-related signaling pathways and increased cellular death (experimental outline and hypothesis of this study are shown in Figure 1).

## Materials and Methods

### Ethical Statement

The use of human material has been approved by the local ethics committee (grant no. 14-101-0189; Ethikkommission, Universität Regensburg; email: ethikkommission@klinik.ukr.de) and written consent of all patients has been obtained.

### Isolation and Culture of Human BMSC and Osteoarthritic Chondrocytes

hBMSC were obtained after femoral head removal from seven patients who underwent total hip arthroplasty surgery (mean age: 64.6 ± 7.8 years, range: 50–72 years, female: 57.1%). hBMSC were harvested by density gradient centrifugation according to established protocols in this lab (Leyh et al., 2014a,b). Subsequently, hBMSC were expanded for three passages in StemMACS Expansion Medium (Miltenyi Biotec, Germany) supplemented with 0.2% MycoZap (#923c1069, Lonza, Switzerland) before usage.

Human articular cartilage biopsies were obtained from OA patients' knee joints who underwent total knee arthroplasty surgery (mean age: 67.3 ± 7.7 years, range: 57–79 years, female: 87.5%). Cartilage was cut into small pieces after being removed from the subchondral bone and digested with 0.2% type II collagenase in Dulbecco's modified Eagle's medium (DMEM F12) (#D8437, Sigma-Aldrich, Germany). After 18 h digestion, passage 0 chondrocytes were pelleted and expanded in DMEM F12 medium, supplemented with 10% fetal calf serum (FCS) (#F7524, Sigma-Aldrich, Germany) and 1% penicillin–streptomycin (#A5955, Sigma-Aldrich, Germany).

### FCS EV Depletion and Characterization

In order to deplete the FCS from EVs, FCS was diluted in α-MEM medium (M8042, Sigma-Aldrich, Germany) to different concentrations (20, 50, and 100%) and subjected to ultracentrifugation at 120,000 × g (L-90 K, Beckman Coulter, Brea; USA) at 4°C for 18 h. Phosphate-buffered saline (PBS), α-MEM, and commercially available EV-depleted FCS (FCS^depl-com^ #A27208-03, GIBCO, USA) were used as controls. Depletion efficacy was analyzed using western blotting. The comparability of controls and FCS, depleted via ultracentrifugation (FCS^depl-uc^), was verified via proliferation (BrdU incorporation) and apoptosis (caspase 3/7 activity) assays with hBMSC after being cultured for 24 h in the presence of controls and FCS^depl-uc^.

### hBMSC EV Isolation

Differential centrifugation and ultracentrifuge steps were applied to pellet EVs from hBMSC-conditioned culture supernatant according to protocols previously published (Théry et al., 2006). Briefly, we applied sequential centrifugation steps for 10 min at 300 × g to remove cells, followed by 10 min at 2,000 × g to remove dead cells, and finally for 30 min at 10,000 × g to remove cell debris. This procedure was followed by ultracentrifugation (Optima L-90K Ultracentrifuge, SW32-Ti rotor, Beckman Coulter, Brea, USA) of the supernatant at 120,000 × g for 70 min at 4°C and resolving of the pellet in PBS. Finally, the EVs were pelleted at 120,000 × g for 70 min at 4°C, and the pellet was resuspended in the presence of 25 mM trehalose (#8897.1, ROTH, Germany) in PBS and stored at −80°C for following experiments. Protein concentration of EV preparations was examined by a BCA Protein Assay Kit according to manufacturer's protocol (#23227, Thermo Scientific, Rockford; USA).

### hBMSC Labeling and EV Uptake Test Using PKH26

According to manufacturer's protocol, 1 × 10^7^ hBMSC were labeled with PKH26 Red Fluorescent Cell Linker Mini Kit for general cell membrane labeling (Sigma-Aldrich, Steinheim, Germany). For the control samples (to control for false positives), PBS was used accordingly. Subsequently, the PKH26-labeled hBMSC were cultured in α-MEM, supplemented with 10% FCS^depl-uc^ and 1% penicillin–streptomycin. PKH26-labeled hBMSC-derived EVs were collected and purified as described under the *hBMSC EV Isolation* section. In eight-well chamber slides (#354118, Falcon, USA) with DMEM F12 medium (supplemented with 10% FCS and 1% penicillin–streptomycin), 1 × 10^4^ OA-CH were seeded. After 12 h, cells were incubated with purified PKH26-labeled hBMSC EVs at a concentration of 10 ug/ml for another 12 h, and subsequently, OA-CH were stained with phalloidin (#ab235137, Abcam, UK) and DAPI (#D3571, Thermo Fisher, Germany). Internalization of PKH26-labeled hBMSC EVs was visualized and documented with fluorescence microscopy (#BX61, OLYMPUS, Japan).

In order to determine whether the hBMSC-EVs could be uptaken by cartilage explants *in situ*, hBMSC-EVs and PBS (negative control) were labeled with PKH26, then used for incubation of cartilage explants (obtained from an OA patient who underwent total knee arthroplasty) for 1 to 5 days. Briefly, cartilage biopsies were cut into small pieces (~0.5 cm × 0.5 cm) after being removed from the subchondral bone, cultured with DMEM F12 medium (supplemented with 10% FCS and 1% penicillin–streptomycin). After 24 h, the medium was changed to a medium supplemented with PKH26-hBMSC-EVs (10 μg/ml) or the same volume of PKH26-PBS. Incubation was stopped at different time points (1, 2, 4, and 5 days). Subsequently, cartilage samples were placed in 4% paraformaldehyde for 24 h for fixation, followed by a conventional paraffin embedding. The wax blocks were cut into 6 μm paraffin sections. After removing paraffin using Histol (#6640.1, ROTH, Germany) and different concentrations of highly pure ethanol (100, 96, 70, and 50%; #5054.4, ROTH, Germany), samples were stained with DAPI (#D3571, Thermo Fisher, Germany) for 15 min in the dark, and subsequently, photos were taken with a fluorescence microscope (#BX61, OLYMPUS, Japan).

### IL-1β Stimulation of OA Chondrocytes and hBMSC-EV Treatment

In six-well plates with DMEM F12 medium (supplemented with 10% normal FCS and 1% penicillin–streptomycin), 2 × 10^5^ OA-CH (passage 2–4) were cultured, stimulated with IL-1β (1 ng/ml) (MAN0004230, Thermo Fisher, USA) for 24 h, and incubated with hBMSC-EVs (10 ug/ml) for 24 h before being prepared for the following experiments.

### RNA Extraction and Real-Time PCR Analysis

Total RNA of cells was isolated using the Absolutely RNA Miniprep Kit (Agilent Technologies, USA) according to the manufacturer's instructions and reverse-transcribed into cDNA using AffinityScript QPCR cDNA Synthesis Kit (#600559, Agilent Technologies, USA). Subsequently, real-time PCR for mRNA expression was performed using Brilliant III Ultra-Fast SYBR® Green QPCR Master Mix (#600882, Agilent Technologies, USA) using a MX3005P QPCR System (Agilent Technologies, Santa Clara, USA). All genes were analyzed relatively, calibrated to the expression of control cell culture groups, and normalized to GAPDH and TATA-binding protein (TBP). Sequences of primers used for real-time PCR in this study are listed in Table 1.

**Table 1:** Primer sequences for qPCR.

**Gene**	**Primer sequence**
ACAN	Fwd: 5′-CTATACCCCAGTGGGCACAT-3′
	Rev: 5′-GGCACTTCAGTTGCAGAAGG−3′
BCL2	Fwd: 5′-ATGTGTGTGGAGAGCGTCAA-3′
	Rev: 5′-ACAGTTCCACAAAGGCATCC-3′
COMP	Fwd: 5′-AGGGAGATCGTGCAGACAA-3′
	Rev: 5′-AGCTGGAGCTGTCCTGGTAG-3′
COL2A1	Fwd: 5′-CCAGATGACCTTCCTACGCC-3′
	Rev: 5′-TTCAGGGCAGTGTACGTGAAC-3′
SOX9	Fwd: 5′-GTACCCGCACTTGCACAAC-3′
	Rev: 5′-TCTCGCTCTCGTTCAGAAGTC-3′
PRG4	Fwd: 5′-CATGGAGTGCTGCCCTGATT-3′
	Rev: 5′-TCTTACATTGGGCGTCGCAG-3′
COLX	Fwd: 5′-CAC GTT TGG GTA GGC CTG TA-3′
	Rev: 5′-TCT GTG AGC TCC ATG ATT GC-3′
ALPL	Fwd: 5′-CCTCCTCGGAAGACACTCTG-3′
	Rev: 5′-AGACTGCGCCTGGTAGTTGT-3′
IL1B	Fwd: 5′-TAAGCCCACTCTACAGCTGG-3′
	Rev: 5′-GAGAGGTGCTGATGTACCAG-3′
MMP13	Fwd: 5′-GACTGGTAATGGCATCAAGGGA-3′
	Rev: 5′-CACCGGCAAAAGCCACTTTA-3′
TBP	Fwd: 5′-TTG TAC CGCAGCTGCAAA AT-3′
	Rev: 5′-TAT ATT CGG CGT TTC GGG CA-3′
GAPDH	Fwd: 5′-CTGACTTCAACAGCGACACC-3′
	Rev: 5′-CCCTGTTGCTGTAGCCAA AT-3′

### Nanoparticle Tracking Analysis

Nanoparticle tracking analysis (NTA) was applied to measure the distribution of particle size and concentration in EV preparations using a NanoSight NS300 (Malvern Instruments, Malvern, UK). The accuracy of NTA was confirmed with 100-nm polystyrene beads (Sigma-Aldrich, Germany) immediately before measurements. EV samples were diluted 1:100 in PBS, measurements performed at 25°C, and five measurements of 30 s were recorded for each EVs sample.

### Scanning Electron Microscopy

The EV samples were fixed with 2.5% glutaraldehyde (Sigma-Aldrich, Germany) in H_2_O for 30 min. After washing twice with D2-H2O (#10977-035, Thermo Fisher, Germany), the fixed EVs were dehydrated using differential concentrations of highly pure ethanol (30, 50, 70, 80, 90, 96, and 100%; #5054.4, ROTH, Germany). Samples were collected in 50 μl 100% ethanol and pipetted onto tissue culture coverslips (#83.1840.002; Sarstedt, Nümbrecht, Germany). Then, 200 μl absolute EtOH was added, and the sample was processed in a critical point dryer and sputtered with gold-platinum. Imaging was performed after 2 h with an acceleration voltage of 3 kV, spot size of 2, 5–6 mm distance, and an aperture stop of 30 μm using a FEI Quanta 400 FEG (FEI, Frankfurt a. Main, Germany).

### Protein Extraction and Western Blot Analysis

hBMSC and OA-CH were washed two times with PBS and lysed with RIPA buffer (Thermo Scientific, Waltham, MA) containing phosphatase and proteinase inhibitors (Roche, Germany). The concentration of cellular protein and EVs was quantified using a BCA protein kit assay (see *hBMSC EV Isolation*). Cell lysates (10 ug) or EVs (10 ug) were mixed with SDS-sample loading buffer (#B7053, Sigma-Aldrich, Germany), followed by boiling for 5 min at 95°C, and subjected to 12% SDS-PAGE. The proteins were transferred to 0.22 μm PVDF membranes (Roche, Penzberg, Germany) after electrophoretic separation. Blot membranes were blocked with 5% BSA for 1 h at room temperature and incubated with primary antibodies on a shaker overnight at 4°C. After washing, the membranes were incubated with the appropriate horseradish peroxidase-coupled secondary antibodies (Santa Cruz Biotechnology and Jackson ImmunoResearch, West Grove, PA). Proteins were examined using ECL detection reagents (Thermo Scientific; Germany), and signals from cell lysates were normalized to β-actin (except for EV band analysis). Antibodies used for this study are listed in Table 2.

**Table 2:** Primary antibodies for western blot.

**Primary antibodies to**	**Company and catalog no**.	**Dilution for western blotting**
CD 9	Thermo Fisher: 10626D	1:1,000
CD 81	Thermo Fisher: 10630D	1:500
CD 63	Thermo Fisher: 10628D	1:1,000
Phospho-P38	Cell signaling: 4511	1:1,000
P38	Cell signaling: 9212	1:1,000
Phospho-Erk1/2	Cell signaling: 4370	1:1,000
Erk1/2	Cell signaling: 4695	1:1,000
Phospho-Akt	Cell signaling: 4060	1:1,000
Akt	Cell signaling: 4691	1:1,000
Phospho-NF-κB p65	Cell signaling: 3033	1:1,000
NF-κB p65	Cell signaling: 8242	1:1,000
Phospho-TAK1	Cell signaling: 4508	1:1,000
TAK1	Cell signaling: 5206	1:1,000
β-actin	Abcam: ab8227	1:10,000

### BrdU Incorporation Assay

BrdU–enzyme-linked immunosorbent assay (ELISA)-based assay (Roche, Penzberg, Germany) was performed to determine cell proliferation according to the manufacturer's protocol. Into 96-well plates, 5 × 10^3^ hBMSC or OA-CH were seeded. After 24 h culturing, culture DMEM F12 medium (supplemented with 10% FCS and 1% penicillin–streptomycin) was exchanged to medium containing BrdU and appropriate treatment agents. After an additional 24 h, the amount of BrdU incorporated into the cells was determined by binding of a mouse anti-BrdU antibody conjugated with horseradish peroxidase. After color development, the signal was monitored at 450/690 nm with a Tecan ELISA reader (Mannedorf, Switzerland).

### Caspase-3/7 Assay

Caspase-3/7 enzymatic activity was quantified to indicate cell apoptosis using an Apo-ONE Homogeneous Caspase-3/7 assay kit (#G7791, Promega Corporation, Madison; USA) according to manufacturer's instructions. A non-fluorescent caspase substrate (Z-DEVDR110), added to the 1 × 10^4^ hBMSC (cultured in StemMACS Expansion Medium, supplemented with 0.2% MycoZap) or OA-CH (cultured in DMEM F12 medium, supplemented with 10% FCS and 1% penicillin–streptomycin) seeded in 96-well plate, was cleaved into fluorescent molecules with an emission maximum at 521 nm and evaluated with a Tecan ELISA reader (Mannedorf, Switzerland).

### Live and Dead Cell Staining Assay

Calcein-AM (#17783-1MG, Sigma, USA) and ethidium homodimer (46043-1MG-F, Sigma, USA) were utilized for simultaneous fluorescence detection. In 24-well plates, 2 × 10^4^ hBMSC or OA-CH were seeded for 24 h, before Calcein-AM (3 μM) and ethidium homodimer (2 μM) were added to the culture supernatant according to manufacturer's protocols. The live (green) and dead (red) cells were determined using a fluorescence microscope (#Eclipse TE2000-U, Nikon, Japan) and ImageJ software (National Institutes of Health, USA).

### Migration (Wound Healing) Assay

In cell culture inserts, 1 × 10^4^ OA-CH (passage 2–4) were cultured (#80206, ibidi, Germany) until reaching 100% confluency. Subsequently, cell culture inserts were removed, leaving a gap to be filled by migrating cells. Cells were washed two times with PBS, and after changing medium to 1% FCS, IL-1β (1 ng/ml) and/or hBMSC-EVs (10 μg/ml) were added. Pictures were taken under a light microscope (#Eclipse TS100, Nikon, Japan) after 0, 24, 48, and 72 h post wounding.

### Immunofluorescence Analysis of Phosphorylated Signaling Molecules

Immunofluorescence analysis was performed to determine the phosphorylation status of OA-CH MAPK, PI3K/AKT, and NFκb pathway components. Briefly, 1 × 10^4^ OA-CH were seeded in eight-well chamber slides (#354118, Falcon, USA) with DMEM F12 medium (supplemented with 10% normal FCS and 1% penicillin–streptomycin). Afterwards, cells were incubated with IL-1β (1 ng/ml) and/or hBMSC-EVs (10 μg/ml). After fixation and blocking according to the manufacturer's instruction, cells were incubated with appropriate primary antibodies (Phospho-p38, 1:1,600, Cell Signaling; Phospho-Erk, 1:200, Cell Signaling; Phospho-AKT, 1:400, Cell Signaling, Germany; Phospho-NF-κB p65, 1:1,600, Cell Signaling) overnight at 4°C. After rinsing with PBS, OA-CH were incubated with appropriate secondary antibodies (Alexa Fluor® 488 Conjugate, #4412, Cell Signaling, Germany). Then, OA-CH were stained with DAPI (#D3571, Thermo Fisher, Germany) and Alexa Fluor™ 568-conjugated phalloidin (#A12380, Thermo Fisher, Germany) as previously described (Gorse and Lafrenaye, 2018). OA-CH cell shape was visualized with fluorescence microscopy (#BX61, OLYMPUS, Japan). The phosphorylation level of signaling pathway components was calculated via counting the number of positively stained OA-CH (green). The total number of OA-CH and those that stained positive for phosphorylated p38, ERK, Akt, and NF-κB were counted in three random areas of each well using ImageJ software (National Institutes of Health, USA). The percentage of positive stained OA-CH for the respective antigen and the relative fold change of the different antigen groups were determined.

### Statistical Analysis

All the data were expressed as the mean ± SD. Statistical analysis was performed using Prism8.02 software (GraphPad Software, USA). Each assay was performed in replicates and repeated in at least three–four independent experiments. One-way ANOVA with Newman–Keuls Multiple Comparison Test were used to compare the results in each experiment. A value of *P* < 0.05 was regarded as statistical significant.

## Results

### hBMSC-EV Isolation and Characterization

**Figure 2:**
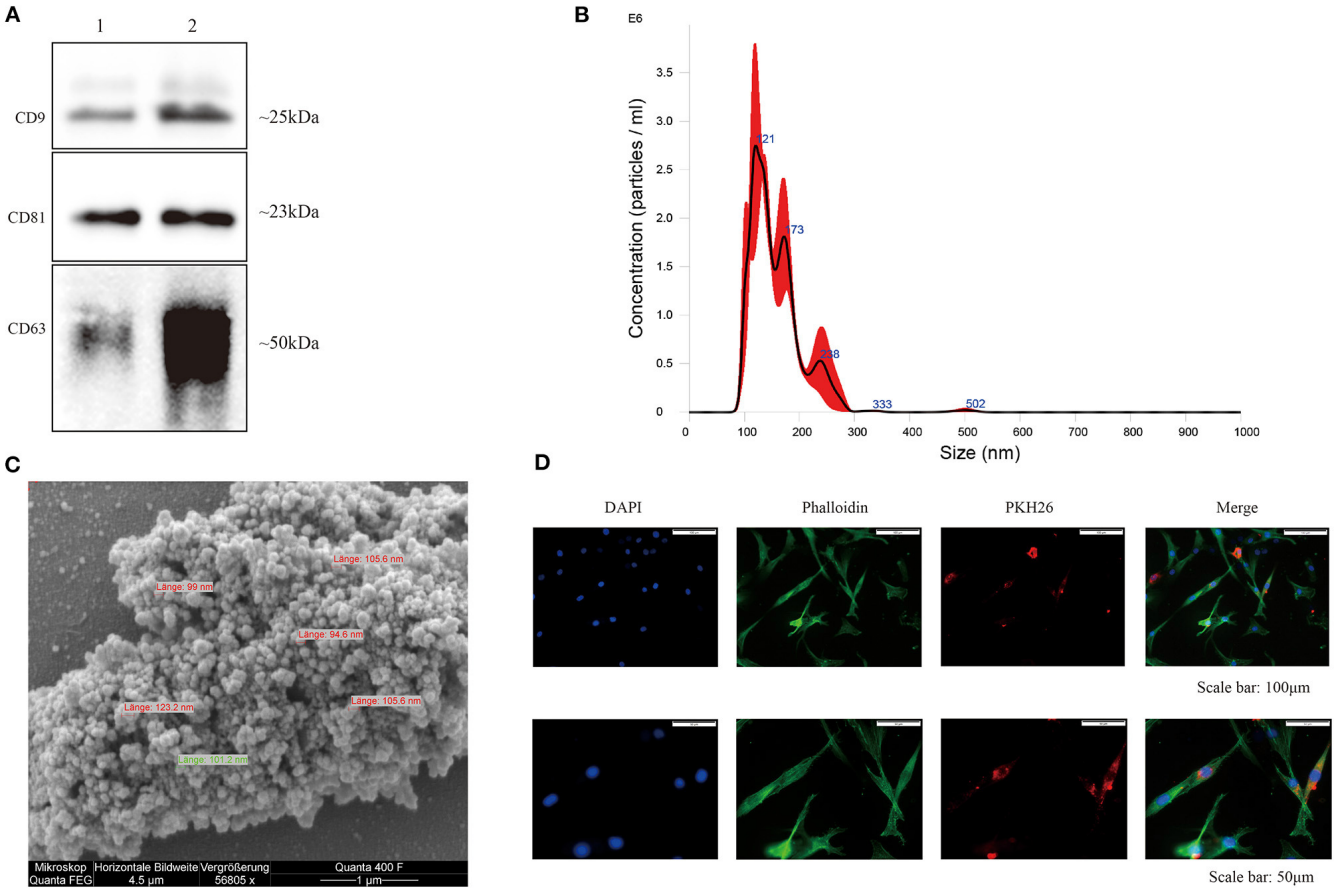
Characterization of hBMSC-derived EVs. **(A)** Representative western blot image (*n* = 4) demonstrates standard surface markers (CD9, CD63, and CD81) of hBMSC-derived EVs (lane 1: hBMSC lysate; lane 2: hBMSC-EVs). **(B)** Particle size distribution of hBMSC-EVs was measured by NTA. **(C)** Morphology of hBMSC-EVs was monitored by SEM, scale bar: 1 μm. **(D)** Cell nuclei were stained with DAPI (blue) and chondrocytes were stained with phalloidin (green) to visualize the cytoskeleton. PKH26-labeled hBMSC-derived EVs (red) internalized by chondrocytes were visualized with fluorescent microscopy.

EVs were isolated from hBMSC-conditioned medium, and classical surface makers (CD9, CD63, and CD81) of EVs derived from hBMSC and OA-CH were verified by western blotting (Figure 2A and Supplementary Figure 1). Particle size distribution of hBMSC-EVs was measured by NTA, which revealed the average size of particles as a mean of 124.6 nm (SD ± 81.1 nm) corresponding to the standard size of EVs (Figure 2B). Morphology of hBMSC-EVs was monitored by SEM, which revealed particles with an average diameter of 90–120 nm corresponding to NTA data (Figure 2C). As it is not trivial to find single, non-aggregated EVs with the SEM technique, we used highly concentrated EV samples. For usage of EVs in functional assays, we are adding 25 mM trehalose in PBS to the EV solution to prevent EVs from aggregating and resuspended the EV samples before usage. Supplementary Figure 2 presents a SEM image of EVs less aggregated.

In order to evaluate internalization of hBMSC-EVs into OA-CH, OA-CH were incubated with PKH26-labeled hBMSC-EVs for 12 h. A strong intracellular fluorescence signal (red) was detected in OA-CH cytoplasm indicating that hBMSC-EVs were uptaken by OA-CH (Figure 2D). Furthermore, to analyze if EVs can penetrate the cartilage matrix, we treated cartilage explants with PKH26-hBMSC-EVs and PKH26-PBS (negative control) for 1 to 5 days. Red fluorescence, indicating presence of PKH26-labeled EVs, can be seen around the chondrocyte nuclei in the EV-treated groups; in contrast, in the PBS control group, there was no fluorescence around the chondrocyte nuclei visible (Supplementary Figure 3). These data suggested that we have successfully isolated and purified EVs from hBMSC culture supernatant, which can be internalized by OA-CH and cartilage explants *ex vivo*.

### FCS EV Depletion and Characterization

**Figure 3:**
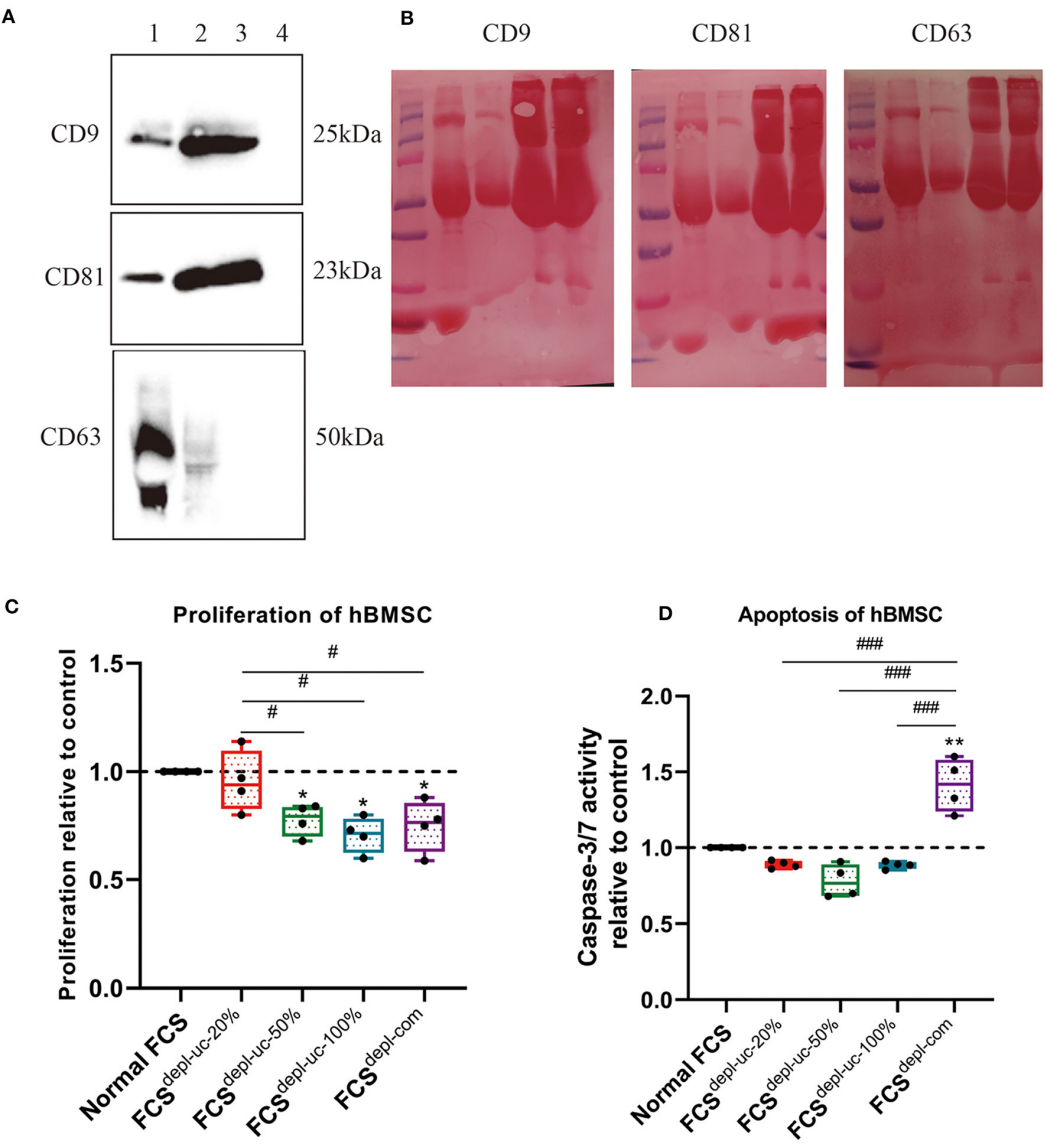
Characterization of EV-depleted FCS (FCS^depl-uc^). Prior to ultracentrifugation, normal FCS was diluted in culture medium to different concentrations (20, 50%, and undiluted = 100%); different FCS^depl-uc^ groups were identified after ultracentrifugation. **(A,B)** FCS^depl-uc-20%^ was controlled for EV surface makers (CD9, CD63, and CD81) detected by western blotting (lane 1: hBMSC lysate; lane 2: hBMSC-EVs; lane 3: undepleted FCS; lane 4: FCS^depl-uc-20%^). Representative western blot image **(A)** and Ponceau Red-stained images for each surface marker **(B)** are shown; *n* = 3. **(C,D)** Proliferation and apoptosis of hBMSC were determined by BrdU assay and caspase-3/7 activity assay separately after being incubated in culture medium supplemented with the different FCS groups for 24 h. All values represent mean ± standard deviation. *Significant difference to control: *p < 0.05; **p < 0.01; ^#^Significant difference between groups: ^#^*p* < 0.05; ^###^*p* < 0.001 one-way ANOVA with Newman–Keuls Multiple Comparison Test; *n* = 4.

In order to deplete EVs from FCS, ultracentrifugation was performed for 18 h after FCS was diluted with α-MEM to different concentrations (20 and 50%) or kept undiluted (100%). PBS, α-MEM medium without FCS, and commercial EV-depleted FCS (FCS^depl-com^) served as negative controls. Our FCS^depl-uc^ (FCS^depl-uc-20%^, FCS^depl-uc-50%^, and FCS^depl-uc-100%^) was analyzed via western blotting for classical EV marker proteins (CD9, CD81, and CD63). These surface markers were detected in hBMSC lysates and hBMSC-EVs, while FCS^depl-uc-20%^, undergoing the same ultracentrifugation procedure and applied in similar quantities for western blot analysis, was negative. This was in contrast to the normal non-depleted FCS confirming the efficiency of our EV depletion strategy (Figures 3A,B). After 24 h of culturing with the different FCS groups (final concentration of FCS in cell culture was always 10%), proliferation of hBMSC, which were cultured using FCS^depl-uc-20%^ and undepleted FCS, was significantly higher than the other two EV-free FCS groups, but no significant difference to the undepleted FCS group was detected (Figure 3C). Notably, commercial EV-depleted FCS^depl-com^ induced higher apoptosis in hBMSC compared to undepleted FCS and FCS^depl-uc^ groups (Figure 3D).

### EV-Induced Migration of IL-1ß-Treated OA-CH

**Figure 4:**
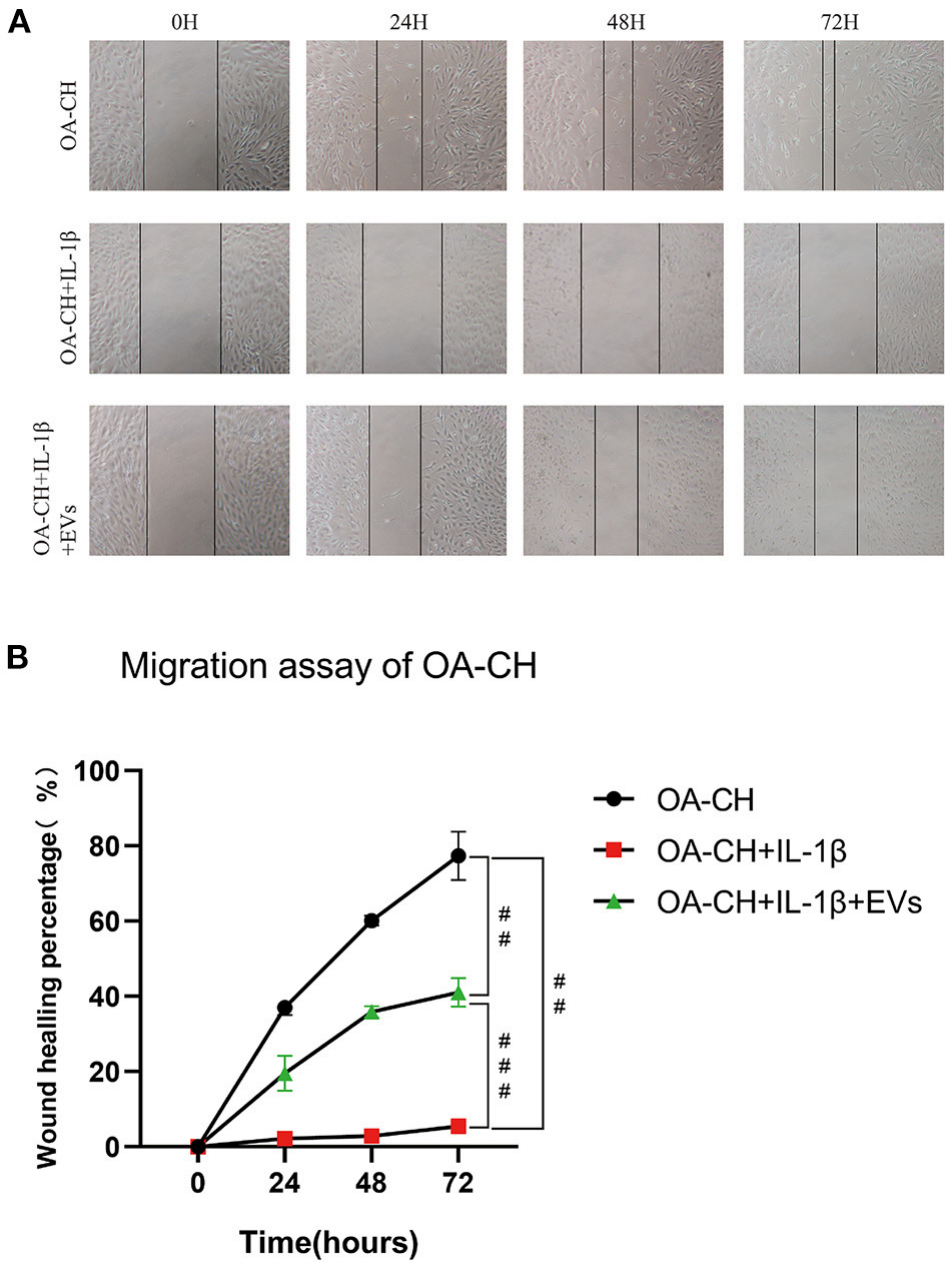
Effect of hBMSC-EVs on migration of OA-CH and IL-1β-induced OA-CH. **(A)** A scratch (wound healing) assay was used to evaluate the effect of hBMSC-EVs on migration of IL-1β-induced OA-CH. Pictures of gaps were taken 0, 24, 48, and 72 h after treatment of cells with EVs and IL-1β. **(B)** Gap closure percentage was used to calculate the migration ability of each group. The OA-CH group without treatment is used as migration control (black bars); ^#^*p* < 0.05; ^##^*p* < 0.01; ^###^*p* < 0.001. One-way ANOVA with Newman–Keuls Multiple Comparison Test; *n* = 3.

A scratch (wound healing) assay was used to analyze the effect of hBMSC-EVs on migration of IL-1β-treated OA-CH. Treatment with IL-1β reduced wound-healing capacity of OA-CH almost completely (middle row; Figure 4A). We observed the highest wound-healing percentage in the OA-CH control group whereas the IL-1β^+^ EV group was in between (upper and lower panel; Figure 4A). Quantification of cell culture data reveals that addition of EVs significantly induced wound-healing capacity, i.e., migration of IL-1β-treated OA-CH compared to Il-1β-treated OA-CH without EVs (Figure 4B).

These results indicate that EVs can restore migration capacity of IL-1β-treated OA-CH.

### EVs Promoted Proliferation and Inhibited Apoptosis of OA-CH and IL-1β-Treated OA-CH

**Figure 5:**
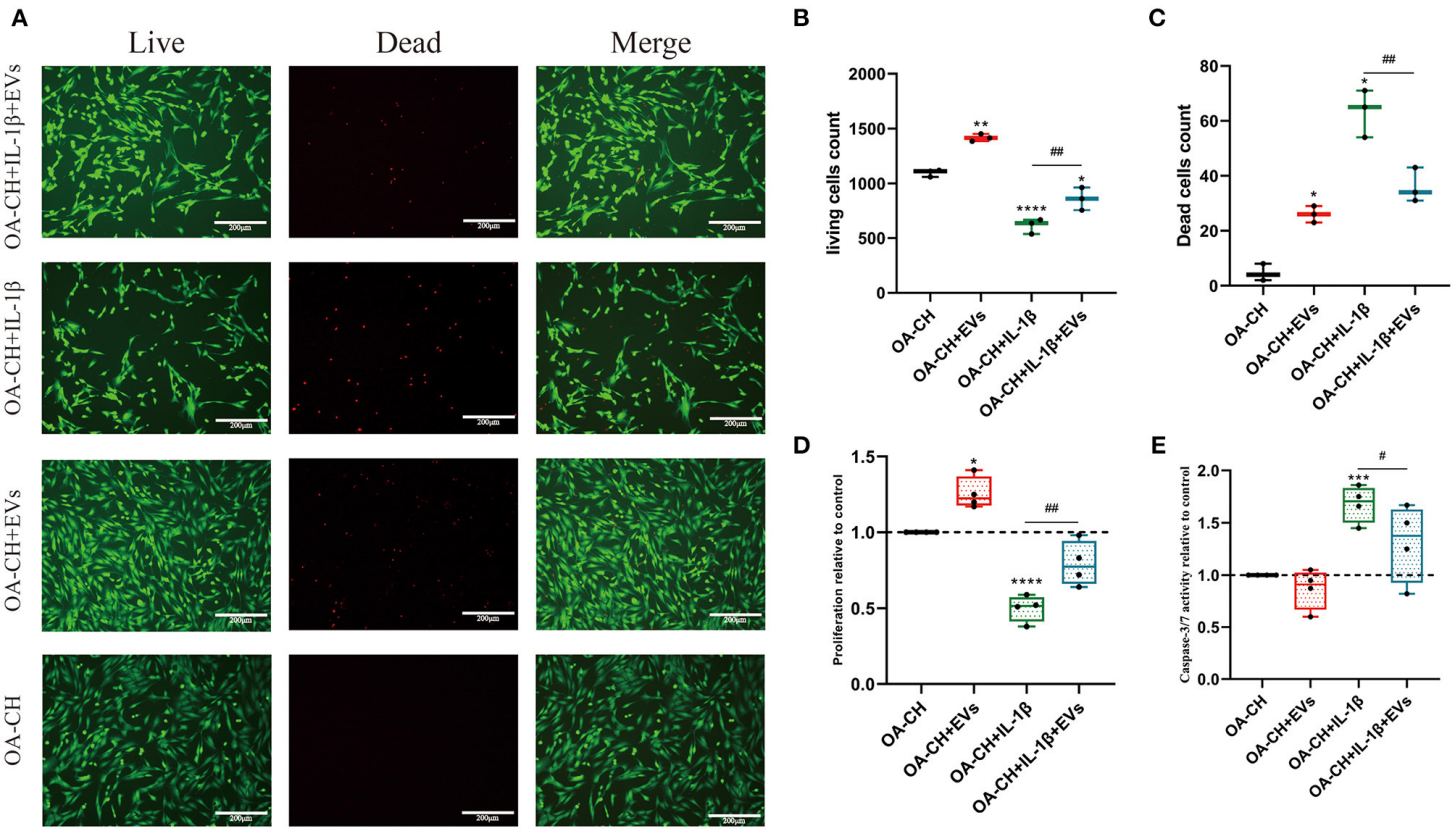
Effect of hBMSC-EVs on vitality, proliferation, and apoptosis of OA-CH and IL-1β-induced OA-CH. **(A–C)** Live and dead cells were visualized by fluorescence microscopy after labeling cells with calcein and ethidium homodimer. Living cells were labeled with calcein (green fluorescence) and dead cells were stained with ethidium homodimer (red fluorescence). Scale bar = 200 μm. **(D,E)** BrdU incorporation assay and Caspase-3/7 activity assay were used to determine the proliferation and apoptosis rate of OA-CH after treatment with hBMSC-EVs or IL-1β for 24 h. The OA-CH group without treatment is used as control and set to 1.0 (black bars). *Significant difference to control (OA-CH): *p < 0.05; **p < 0.01; ***p < 0.001; ****p < 0.001; ^#^significant difference between groups: ^#^*p* < 0.05; ^##^*p* < 0.01; one-way ANOVA with Newman–Keuls Multiple Comparison Test; *n* = 4.

The effects of EVs on OA-CH proliferation and apoptosis were assessed using live and dead cell assay, BrdU incorporation assay, and caspase 3/7 activity assay. Treatment with hBMSC-EVs increased the number of living OA-CH and IL-1β-treated OA-CH significantly. Contrary, dead cell number was significantly decreased in the IL-1β-treated group in the presence of EVs; however, without IL-1β, EVs increased the number of dead OA-CH (Figures 5A–C).

Proliferation of OA-CH and IL-1β-treated OA-CH was significantly higher in the presence of EVs as in groups without EVs (Figure 5D). On the other hand, apoptosis was only decreased in the IL-1β-treated group in the presence of EVs, whereas no difference was observed between both OA-CH and OA-CH^+^ EV groups (Figure 5E). These results suggest that hBMSC-EVs have an anabolic effect on OA-CH and IL-1β-treated OA-CH by promoting proliferation and reducing IL-1β-induced apoptosis.

### Effect of hBMSC-EVs on Gene Expression of OA-CH and IL-1β-Treated OA-CH

**Figure 6:**
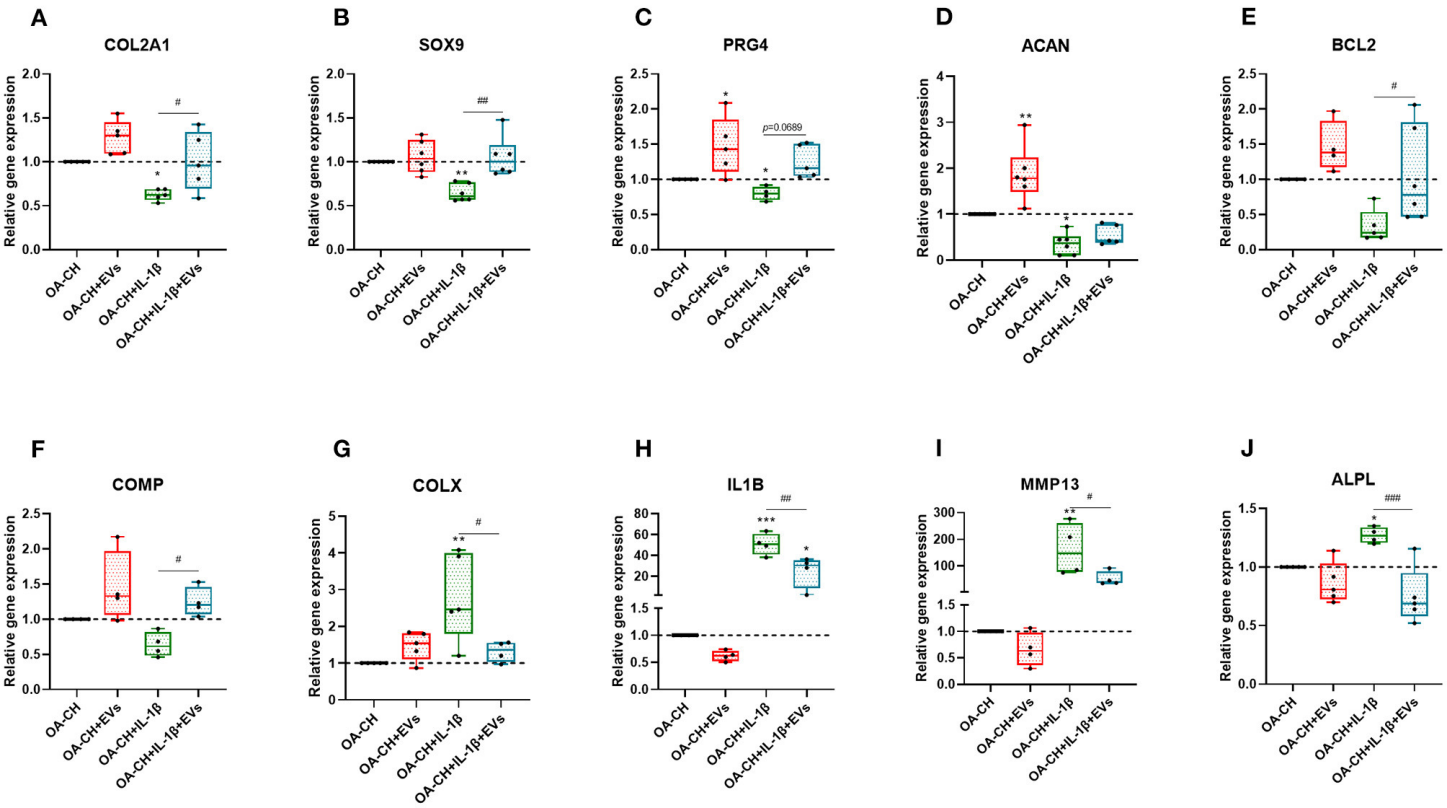
hBMSC-EV effect on gene expression of anabolic and catabolic genes. **(A–F)** After 24 h treatment with IL-1β and/or hBMSC-EVs, gene expression levels of COL2A1, SOX9, PRG4, ACAN, BCL2, and COMP (anabolic) and COLX, IL1B, MMP13, and ALPL (catabolic) were determined using real-time PCR analysis **(G–J)**. All values represent mean ± standard deviation. *Significant difference to control (OA-CH): *p < 0.05; **p < 0.01; ***p < 0.001; ^#^significant difference between groups: ^#^*p* < 0.05; ^##^*p* < 0.01; ^###^*p* < 0.001. One-way ANOVA with Newman–Keuls Multiple Comparison Test (*n* = 4–6); EVs: hBMSC-EVs.

We used qRT-PCR to determine the relative gene expression of anabolic and catabolic markers in OA-CH and IL-1β-treated OA-CH after treatment with hBMSC-EVs. As shown in Figure 6, PRG4, BCL2, and ACAN (aggrecan) gene expression levels were increased in the EV-treated groups compared with control groups without EV (Figures 6C–E). In contrast, gene expression levels of IL1B, ALPL, and MMP13 were decreased (not significant) by trend in the EV groups compared with control groups without EVs (Figures 6H–J). Notably, COL2A1, SOX9, BCL2 ACAN, and COMP gene expression levels were significantly increased in IL-1β^+^ EV groups compared with IL-1β groups without EVs, whereas the gene expression levels of COLX, IL1B, MMP13, and ALPL were significantly decreased in IL-1β^+^ EV groups compared to IL-1β groups without EVs. These results indicated that hBMSC-EVs can promote anabolic gene expression and can inhibit catabolic gene expression in OA-CH in the presence and absence of IL-1β.

### hBMSC-EVs Reduced Phosphorylation of Erk1/2, PI3K/Akt p38, TAK1, and NF-κB of IL-1β-Treated OA-CH

**Figure 7:**
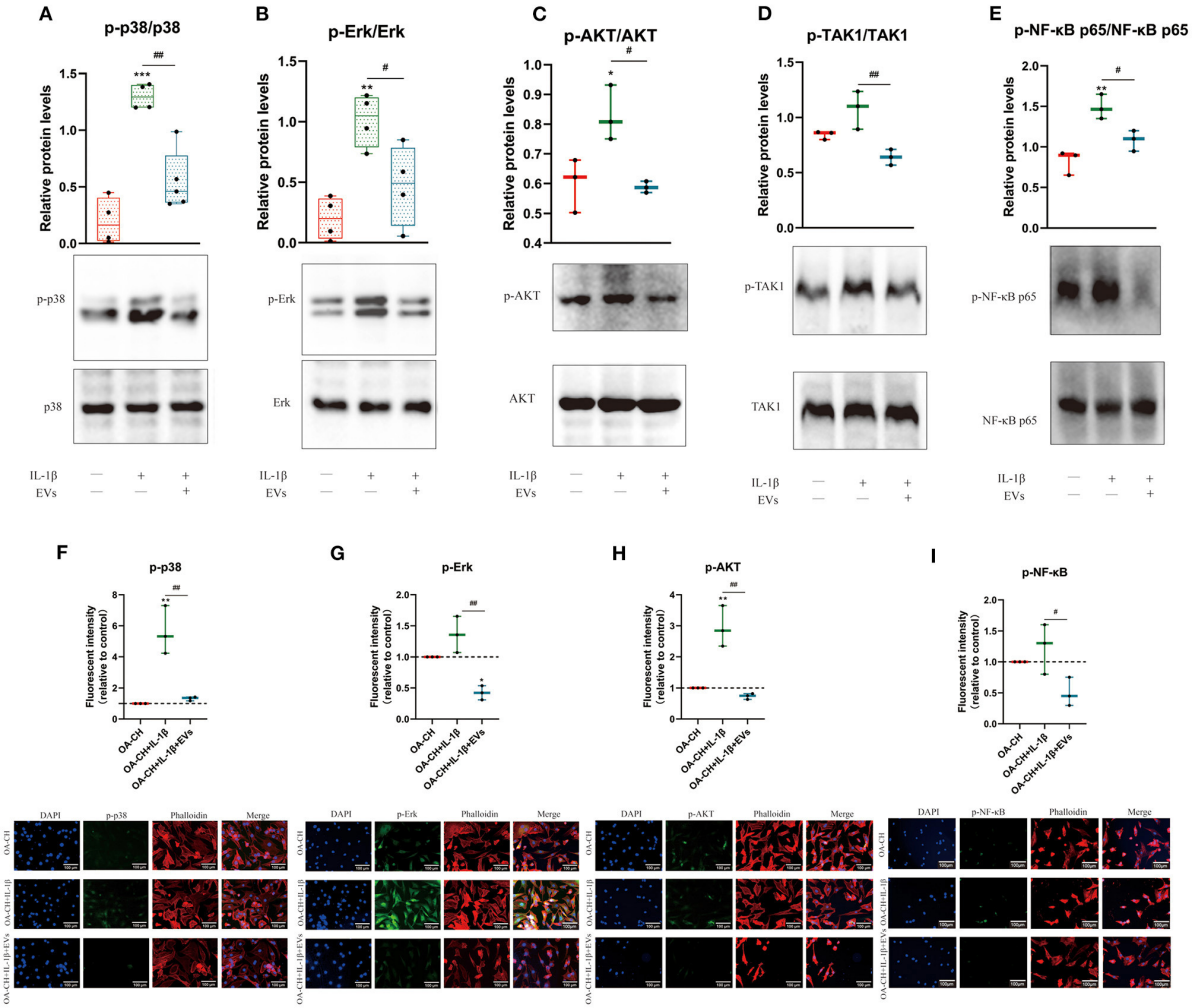
hBMSC-EV effects on IL-1β-induced activation of Erk1/2, PI3K/Akt p38, TAK1, and NF-κB signaling pathways in OA-CH. The phosphorylation levels of Erk1/2, PI3K/Akt, p38, TAK1, and NF-κB in OA-CH were detected by western blotting and immunofluorescence staining. **(A–E)** Quantification of protein phosphorylation level (upper panel) and representative western blot images (lower panel) of Erk1/2, P13K/Akt, p38, TAK1, and NF-κB after 30 min of stimulation with Il-1β and EVs. **(F–I)** Immunofluorescence staining (lower panel) and quantification of protein expression of the phosphorylated forms of Erk1/2, P13K/Akt, p38, and NF-κB (upper panel) in OA-CH after treatment with hBMSC-EVs and/or IL-1β. Scale bar = 100 μm. All values represent mean ± standard deviation. *Significant differences to OA-CH group (control group): *p < 0.05; **p < 0.01; ***p < 0.001; ^#^significant differences between groups: ^#^*p* < 0.05; ^*##*^*p* < 0.01; one-way ANOVA with Newman–Keuls Multiple Comparison Test; *n* = 3.

To analyze the effect of hBMSC-EVs on the activity of pro-inflammatory signaling pathways in IL-1β-treated OA-CH, immunofluorescence and western blotting was performed to evaluate the protein expression and phosphorylation levels of Erk1/2, PI3K/Akt, p38, TAK1, and NF-κB in OA-CH. We detected increased protein levels of p-Erk/Erk, p-AKT/AKT, p-p38/p38, p-TAK1/TAK1, and p-NF-κB/NF-κB in IL-1β-treated OA-CH compared to controls (absence of IL-1β). In IL-1β-treated OA-CH in the presence of EVs, phosphorylation levels of all five kinases were significantly reduced (Figures 7A–E). Furthermore, the immunofluorescence results indicate that the expression of p-p38, p-Erk, p-AKT, and p-NF-κB was upregulated in the IL-1β group and decreased in the IL-1β^+^ EV groups (Figures 7F–I), which is consistent with the western blot results and supports those data by using two different methodological approaches. These results suggested that hBMSC-EVs inhibited IL-1β-induced activation of Erk1/2, AKT, p38, TAK1, and NF-κB signaling pathways in OA-CH.

## Discussion

Accumulating evidence demonstrated that pro-inflammatory cytokines, such as IL-1β, TNF, and IL-6, are involved in the pathophysiology of OA (Robinson et al., 2016; Philpott et al., 2017; Urban and Little, 2018). Especially, IL-1β can act as an independent pro-inflammatory cytokine alone in promoting the progression of OA by inducing chondrocyte apoptosis and pro-inflammatory signaling via MAPK, NF-κB, mTOR, and PI3K/Akt signaling pathways (Moon et al., 2018; Cai et al., 2019; Zhou et al., 2019). Several reports have shown that BMSC-derived EVs play an anti-inflammatory role in OA pathophysiology through regulating MAPK, NF-κB, mTOR, and PI3K/Akt signaling pathways (Zhao et al., 2018; Sun et al., 2019; Xia et al., 2019; Zhu et al., 2019). Vonk et al. (2018) reported that BMSC-derived EVs inhibit the adverse effects of inflammation in TNF-alpha-induced chondrocytes. The present study was designed to investigate putative protective effects of hBMSC-derived EVs on IL-1β-treated articular chondrocytes obtained from OA patients after knee replacement surgery.

In this study, we isolated EVs from hBMSC-conditioned culture supernatant using differential centrifugation and ultracentrifugation steps (Xu et al., 2016; Nguyen et al., 2018). Standard surface EV makers such as CD9, CD63, and CD81 were detected in ultracentrifuged particle isolates by western blotting, indicating that these consist of purified EVs. Additionally, NTA and SEM analysis determined the size of ultracentrifuged particle isolates with a diameter between 90 and 130 nm, indicating that we have prepared EVs in sufficient concentration and purity. Crucial for all following experiments was the proof that chondrocytes were able to internalize hBMSC-derived PKH26-labeled EVs. Notably, not only isolated chondrocytes internalize EVs but also chondrocytes embedded in their ECM are able to internalize EVs, indicating that EVs can penetrate dense extracellular matrices via diffusion.

In order to avoid contamination with exogenous EVs derived from FCS in the culture medium, we prepared EV-depleted FCS before starting with the functional assays. We observed that using 20% FCS for the EV-depletion procedure was most suitable for chondrocyte culture and following functional assays. This finding is consistent with the International Society of Extracellular Vesicles (ISEV) recommended culture protocols (Théry et al., 2018). Of note, the proliferation of OA-CH after being cultured with the FCS^depl-uc−50%^ and FCS^depl-uc−100%^ batches (final concentration of FCS in the culture medium was 10% for experiments) was significantly lower than the FCS^depl-uc-20%^ batch. We assume that critical nutrition supplements of FCS were co-depleted during the ultracentrifugation procedure when using FCS concentrations higher than 20% because the final pellet at the bottom of the tube after ultracentrifugation was highly viscous and difficult to resolve.

Several studies reported that MSC-derived EVs promoted chondrocyte proliferation and inhibited apoptosis (Luo et al., 2019); additionally, some studies demonstrated that EVs derived from MSCs not only promoted regeneration but also attenuated the degeneration of cartilage *in vivo* and *in vitro* (Vonk et al., 2018; Ruiz et al., 2020; Woo et al., 2020). In this study, we observed that hBMSC-EVs promoted proliferation and inhibited apoptosis of OA-CH and IL-1β-stimulated OA-CH. Additionally, our results demonstrated that hBMSC-EVs reversed downregulation of anabolic gene expression (COL2A1, SOX9, PRG4, ACAN, BCL2, and COMP) and upregulation of catabolic gene expression (COLX, IL1B, MMP-13, and ALPL) in IL-1β-stimulated OA-CH. Presumably, this is part of the molecular mechanisms by which hBMSC-EVs can promote proliferation and inhibit apoptosis of OA-CH in the presence and absence of IL-1β. In line with other reports (Liu et al., 2018), our data reveal that EVs derived from hBMSC are able to attenuate the IL-1β-induced catabolic inflammatory effects on OA-CH. With respect to the cargo of EVs, it has been shown that microRNAs (miRs) are potent regulators of chondrocyte metabolism. Mao et al. (2018) investigated the molecular mechanism of EV-associated miR-92a-3p and WNT5A in chondrogenesis and cartilage degeneration. They showed that MSC-miR-92a-3p-derived EVs inhibited cartilage degradation in their murine OA model. The authors suggested that EV-derived miR-92a-3p regulates cartilage development and homeostasis by directly targeting WNT5A. Possibly, EV-derived miR-92a-3p may act as a Wnt inhibitor and exhibits potential as a disease-modifying OA drug. In addition, it was shown that overexpression of miR-140-5p in synovial MSC generated a highly potent version of EVs, which were able to promote chondrocyte proliferation and migration and were able to prevent OA progression in a rat OA model (Tao et al., 2017).

We have observed anti-inflammatory effects of hBMSC-EVs in IL-1β-stimulated OA-CH, but the underlying molecular mechanisms behind these anabolic effects remain unclear. Qi et al. (2019) reported that BMSC-derived EVs inhibited the apoptosis of chondrocytes induced by IL-1β through regulating p38, Erk, and AKT pathways. In order to assess a molecular basis of these anti-inflammatory mechanisms of hBMSC-EVs in IL-1β-stimulated OA-CH, modulation of putative pro-inflammatory signaling pathways was investigated. For that, phosphorylation status of Erk1/2, PI3K/Akt, p38, TAK1, and NF-κB was determined. Our data implicated that hBMSC-EVs can reduce IL-1β-induced phosphorylation of Erk1/2, PI3K/Akt p38, TAK1, and NF-κB and thus reduce the activity of pathways involving these kinases. These findings suggest that the anti-inflammatory effects of hBMSC-EVs might be transduced via modulation of IL-1β-induced pro-inflammatory signaling pathways.

In conclusion, our study demonstrated that hBMSC-EVs alleviated IL-1β-induced catabolic effects on OA-CH via promoting cell proliferation and migration and reducing apoptosis. In addition, catabolic acting genes were downregulated whereas anabolic genes were upregulated in OA-CH in the presence of hBMSC-derived EVs. Our data indicate that molecules contained in these EVs internalized by OA-CH affect the activity of IL-1β-induced pro-inflammatory Erk1/2, PI3K/Akt, p38, TAK1, and NF-κB signaling pathways. According to existing literature, miRs would be strong candidates to be involved in the regulation of these intracellular signaling molecules. Usage of EVs derived from BMSC holds a promising cell-free regenerative intervention strategy avoiding several adverse effects of common cell-based regenerative approaches.

## Data Availability Statement

The original contributions presented in the study are included in the article/Supplementary Material, further inquiries can be directed to the corresponding author/s.

## Ethics Statement

The studies involving human participants were reviewed and approved by Ethikkommission, Universität Regensburg, email: ethikkommission@klinik.ukr.de. The patients/participants provided their written informed consent to participate in this study.

## Author Contributions

SL contributed in the methodology, validation, formal analysis, investigation, data curation, writing, reviewing, and editing the original draft. SS contributed in the conceptualization and writing, reviewing, and editing the manuscript. CL contributed in the methodology and establishment of EV isolation. JG contributed in the methodology and human tissue and cell isolation. MH contributed in the methodology and NTA analysis. MF contributed in the methodology and SEM analysis. SG contributed in the conceptualization, writing, reviewing, editing of the manuscript, project administration, and funding acquisition. All authors have proofread the final version of the manuscript.

## Conflict of Interest

The authors declare that the research was conducted in the absence of any commercial or financial relationships that could be construed as a potential conflict of interest.
