# Supplementary figures and images for: hBMSC-Derived Extracellular Vesicles Attenuate IL-1β-Induced Catabolic Effects on OA-Chondrocytes by Regulating Pro-inflammatory Signaling Pathways
Source: Front Bioeng Biotechnol. 2020 Dec 14;8:603598. doi: 10.3389/fbioe.2020.603598 (PMC7793861; doi:10.3389/fbioe.2020.603598)

a

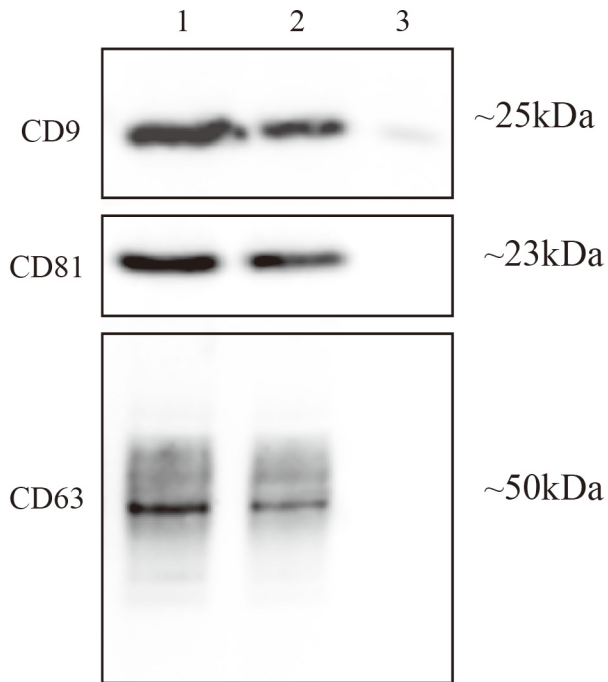

b

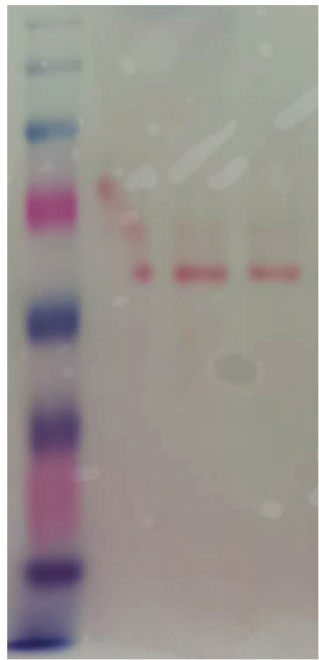

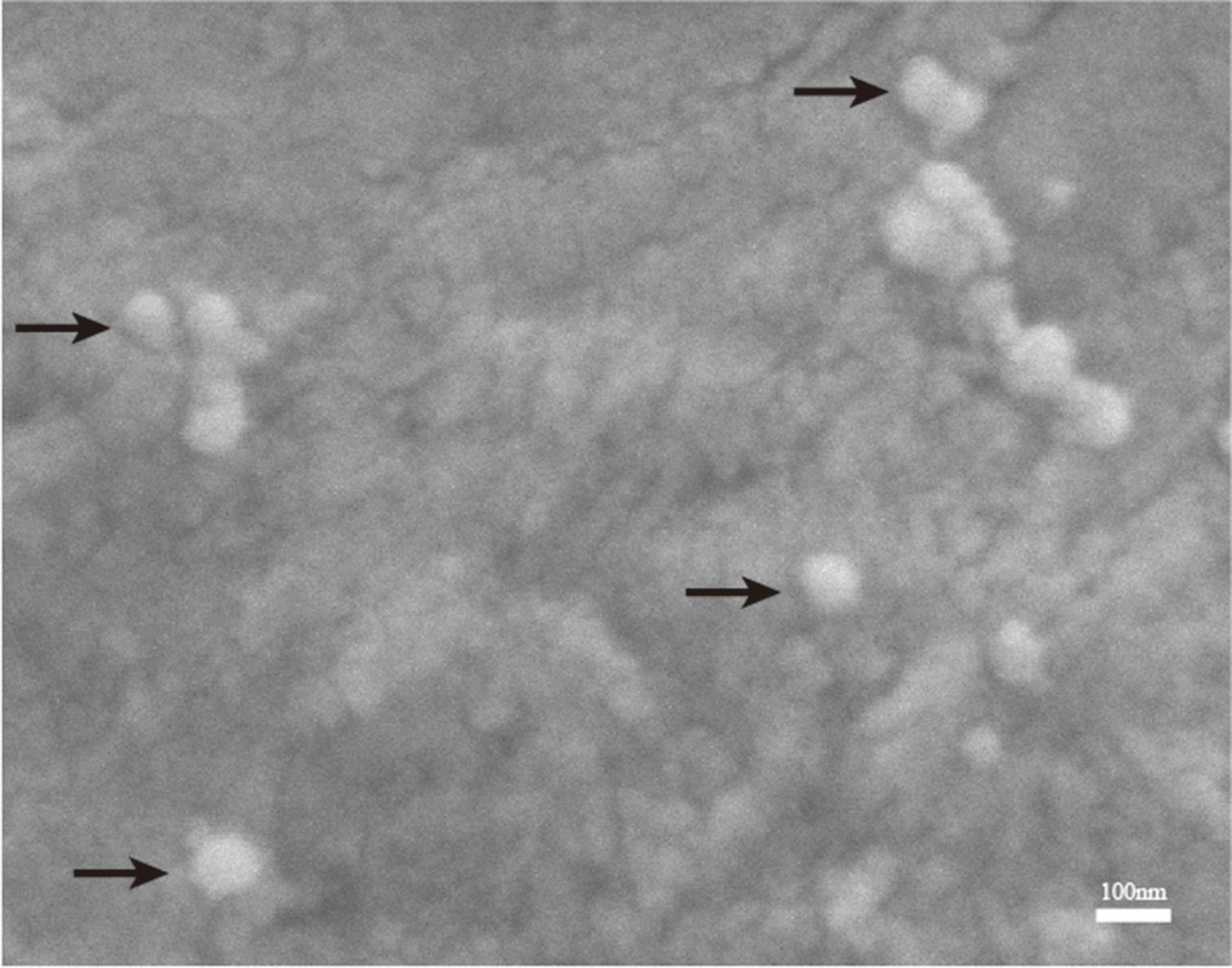

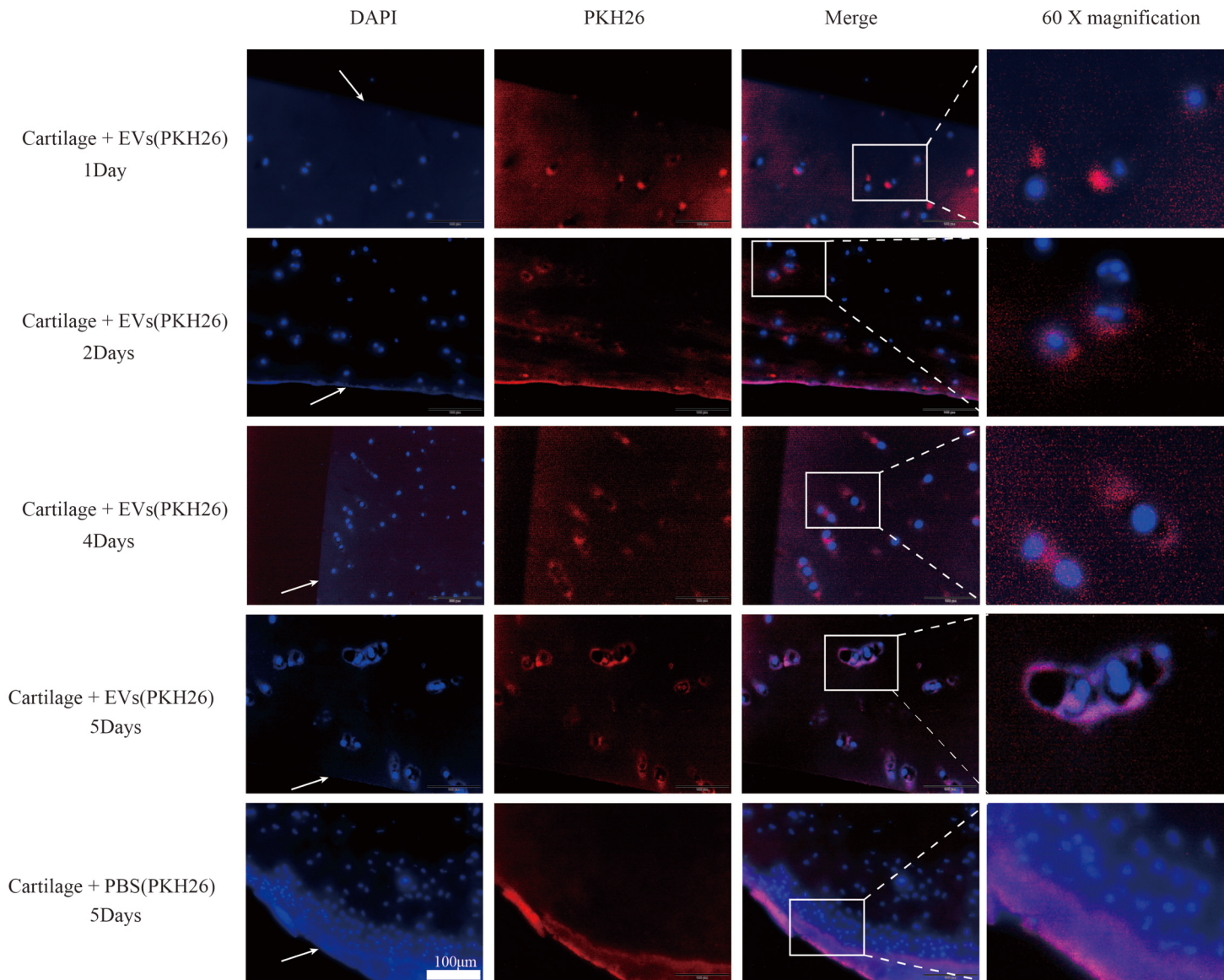

Supplement: Supplementary file 1 [file Data_Sheet_1.PDF]
