# Supplementary material for: hBMSC-Derived Extracellular Vesicles Attenuate IL-1β-Induced Catabolic Effects on OA-Chondrocytes by Regulating Pro-inflammatory Signaling Pathways
Source: Front Bioeng Biotechnol. 2020 Dec 14;8:603598. doi: 10.3389/fbioe.2020.603598 (PMC7793861; doi:10.3389/fbioe.2020.603598)
Supplement: Supplementary file 2 [file Data_Sheet_3.PDF]

Fig. S1 Characterization of EVs depleted FCS

EV surface makers (CD9, CD63, CD81) are detected by western blotting (lane 1: hBMSC lysate; lane 2: EV conditioned medium; lane 3: EVs-depleted conditioned medium). Representative western blot image for each surface marker (a) and respective Ponceau Red stained membrane image (b).

Fig. S2 SEM analysis of EVs

Representative image of not aggregated EVs.

Fig. S3 hBMSC-EVs penetrate the matrix of cartilage explants

In order to determine whether the hBMSC-EVs could be uptaken by cartilage explants in situ, hBMSC-EVs and PBS (negative control) were labeled with PKH26, then were incubation with cartilage explants (obtained from OA patients who underwent total knee arthroplasty) for 1 to 5 days. Samples were stained with DAPI and subsequently photos were taken with a fluorescence microscope.

Red fluorescence, indicating presence of PKH26 labeled EVs can be seen around chondrocytes' nuclei in the EV treated groups, in contrast, in the PBS control group, there was no fluorescence around chondrocytes nuclei visible.

White arrows refer to the surface of cartilage explants.
